# Long-term prognosis in patients with thymoma combined with myasthenia gravis: a propensity score-matching analysis

**DOI:** 10.3389/fmed.2024.1407830

**Published:** 2024-06-14

**Authors:** Kai Zhao, Yiming Liu, Miao Jing, Wenhan Cai, Jiamei Jin, Zirui Zhu, Leilei Shen, Jiaxin Wen, Zhiqiang Xue

**Affiliations:** ^1^Postgraduate School, Medical School of Chinese PLA, Beijing, China; ^2^Department of Thoracic Surgery, Hainan Hospital of Chinese PLA General Hospital, Sanya, China; ^3^Department of Thoracic Surgery, Air Force Hospital of Western Theater Command, PLA, Chengdu, China; ^4^Department of Thoracic Surgery, The First Medical Center of Chinese PLA General Hospital, Beijing, China

**Keywords:** thymoma, myasthenia gravis, prognosis, related factors, propensity score-matching

## Abstract

**Introduction:**

We aimed to assess the impact of myasthenia gravis (MG) on the long-term prognosis in patients with thymoma after surgery and identify related prognostic factors or predictors.

**Methods:**

This retrospective observational study included 509 patients with thymoma (thymoma combined with MG [MG group] and thymoma alone [non-MG group]). Propensity score matching was performed to obtain comparable subsets of 96 patients in each group. A comparative analysis was conducted on various parameters.

**Results:**

Before matching, the 10-year survival and recurrence-free survival rates in both groups were 93.8 and 98.4%, and 85.9 and 93.4%, respectively, with no statistically significant difference observed in the survival curves between the groups (*p* > 0.05). After propensity score matching, 96 matched pairs of patients from both groups were created. The 10-year survival and recurrence-free survival rates in these matched pairs were 96.9 and 97.7%, and 86.9 and 91.1%, respectively, with no statistical significance in the survival curves between the groups (*p* > 0.05). Univariate analysis of patients with thymoma postoperatively revealed that the World Health Organization histopathological classification, Masaoka–Koga stage, Tumor Node Metastasis stage, resection status, and postoperative adjuvant therapy were potentially associated with tumor recurrence after thymoma surgery. Multivariate analysis demonstrated that the Masaoka–Koga stage and postoperative adjuvant therapy independently predicted the risk of recurrence in patients with thymoma after surgery.

**Conclusion:**

There was no difference in prognosis in patients with thymoma with or without MG. The Masaoka–Koga stage has emerged as an independent prognostic factor affecting recurrence-free survival in patients with thymoma, while postoperative adjuvant therapy represents a poor prognostic factor.

## 1 Introduction

Thymomas are relatively uncommon solid chest tumors originating from thymus epithelial cells, with an incidence of approximately 0.13–0.32 cases per 100,000 people annually. It is the most prevalent primary malignant tumor of the anterior mediastinum (1). Owing to its immunological characteristics, many patients with thymoma also present with autoimmune diseases, such as myasthenia gravis (MG), systemic lupus erythematosus, and paraneoplastic glomerulonephritis. Of these conditions, MG is the most common paraneoplastic neurological disease in patients with thymoma, affecting approximately 30–50% of the individuals (2–4).

Surgical resection of thymomas is currently considered an effective treatment for those accompanied by MG (5). In the past, possibly because of limited experience in managing perioperative complications, MG coexistence was regarded as a negative prognostic factor for patients with thymoma. However, recent reports suggest that MG has no detrimental impact on prognosis and may have a favorable effect (6–8). Nevertheless, how MG influences postoperative prognosis in individuals with thymoma remains unclear, given the low incidence rate and long-term follow-up required for enrolled patients. Conducting randomized controlled clinical studies has proven challenging.

Furthermore, non-randomized retrospective clinical studies are often affected by unbalanced confounding factors such as sex, age, and tumor stage. These confounders significantly affect the long-term survival in patients, consequently influencing the accurate interpretation of the study results. To address these issues, this retrospective study aimed to assess MG impact on the long-term prognosis in patients with thymoma after surgery, using propensity score matching (PSM) to balance the distribution of confounding factors among groups and reduce treatment effect bias, and identify related prognostic factors or predictors.

## 2 Materials and methods

### 2.1 Study design and patient selection

The clinical data of 509 patients with thymoma who underwent surgical treatment at the Department of Thoracic Surgery, The First Medical Center, Chinese People’s Liberation Army General Hospital, between January 2010 and October 2023, were retrospectively collected. Patients diagnosed with thymoma based on preoperative chest computed tomography (CT) examination and postoperative pathology, possessing complete clinical and follow-up data, and demonstrating willingness for active telephonic or outpatient follow-up were included. Patients receiving antitumor therapy before surgery, previous thoracic tumor surgery, or those with a history of other malignant tumors were excluded.

**Figure 1:**
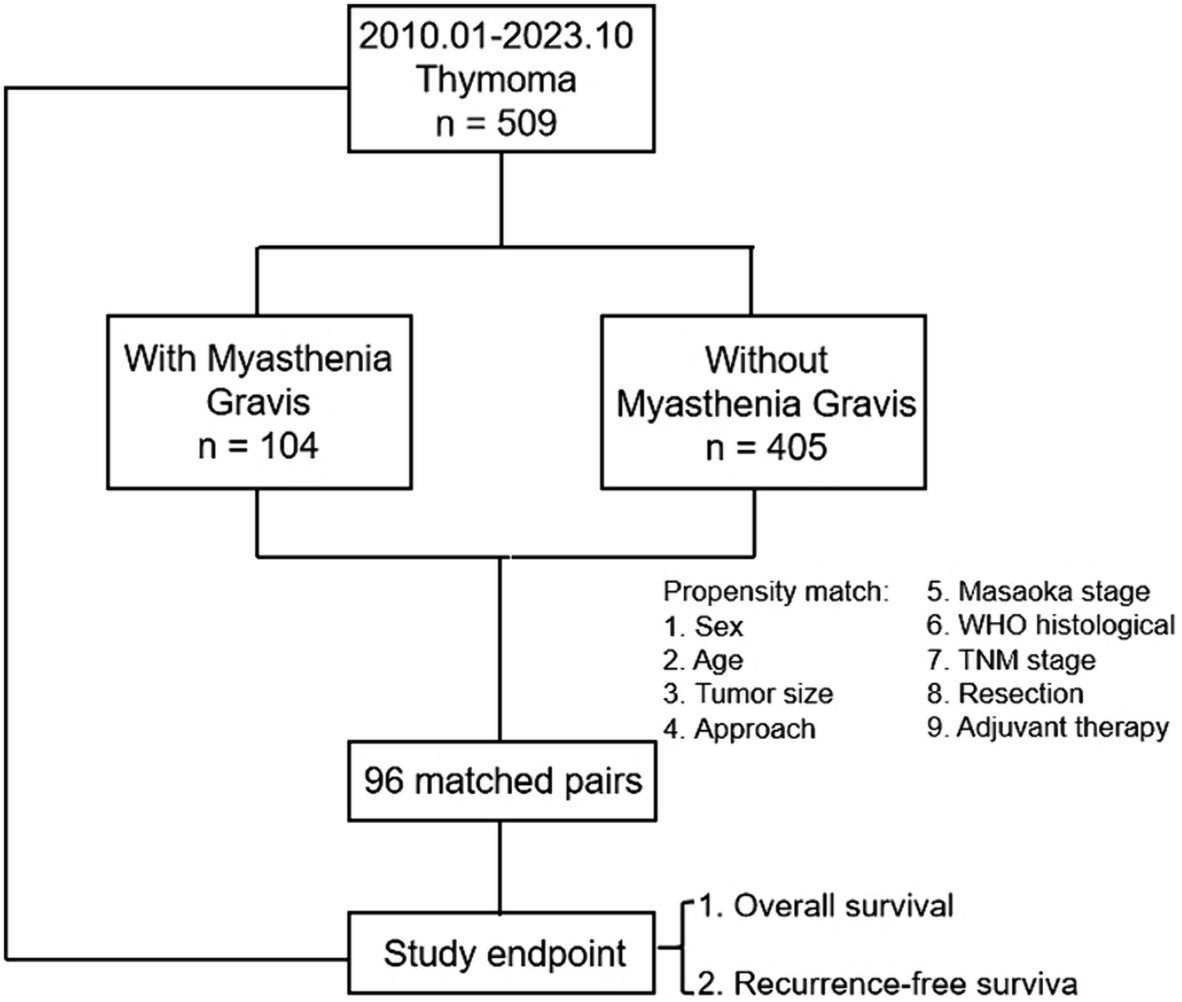
Flow diagram of the study.

Participants were categorized into two groups based on whether they also had MG: thymoma with MG (MG group) and thymoma alone (non-MG group). The diagnostic criteria for thymoma with MG include: (1) presence of typical myasthenic symptoms, demonstrated by a positive fatigue test in the affected muscle; (2) elevated levels of acetylcholine antibodies, positive response to anti-cholinesterase drug test, and confirmation through positive electromyography results; (3) identification of mediastinal mass on chest CT or magnetic resonance imaging scans; and (4) histopathological examination confirming the diagnosis as thymoma. For patients with thymoma combined with MG, efforts have been made to optimize their condition before surgery by alleviating their symptoms and reducing disease progression. A flow diagram of the study is shown in Figure 1.

### 2.2 Observation index and evaluation

The following observation indices were used: sex, age at diagnosis, tumor size, World Health Organization (WHO) histological type, Masaoka–Koga stage, Tumor Node Metastasis (TNM) stage, surgical method, resection status, postoperative adjuvant therapy, recurrence time, and survival time. Thymoma pathological classification was based on the 2015 WHO classification standard (9), and the thymoma was restaged according to the Masaoka–Koga stage (10) and the 9th TNM stage system (11), considering intraoperative findings and postoperative pathology. Surgical methods included minimally invasive thoracoscopic surgery and median sternal incision thoracotomy. The resection status was categorized as R0 for no residual tumor after resection, R1 for microscopic residual tumor, and R2 for locally visible or other features of the residual tumor after resection. In patients with thymoma combined with MG who underwent surgery, the efficacy criteria of MG were evaluated according to the Myasthenia Gravis Foundation of America (MGFA) criteria (12): Complete response indicated no need for medication while not affecting normal work and life; partial response denoted symptom improvement compared to pre-operation, but still requires medication; stable referred to unchanged symptoms and medication dosage from pre-operation; and worsening indicated increased symptoms, dosage, both, or death. Based on the prognostic assessment, patients were classified into the non-response group, comprising those with stable or worsening conditions, and the response group, including those with complete or partial responses.

Follow-up was conducted via outpatient or telephone visits. Follow-up was completed in October 2023, with a median duration of 85 months (1–165 months). Follow-up endpoints included thymoma recurrence, metastasis, or death. Recurrences mainly included local (anterior mediastinal), regional (intrathoracic), and distant (extrinsic) recurrences. Recurrence-free survival (RFS) was defined as the time interval between surgery and the first occurrence of recurrence, whereas overall survival (OS) was defined as the period from the postoperative pathological diagnosis until death from any cause or the end of the follow-up period.

### 2.3 Statistical analysis

Statistical analyses were performed using the SPSS version 26.0 software (IBM Corp., Armonk, Chicago, IL, USA). Measurement data are expressed as means and standard deviations using either a *t*-test or a corrected *t*-test. Count data are expressed as ratios or compositions, and group comparisons were conducted using either the chi-squared or Fisher’s exact test. A nearest neighbor-matching algorithm with a caliper width of 0.02 was employed for one-to-one patient pairing based on propensity scores within a predefined limit. Survival rates were estimated using the Kaplan–Meier method, and differences in survival curves between groups were compared using the log-rank test. Univariate and multivariate analyses were performed using a Cox proportional hazards model. Statistical significance was set at *p* < 0.05.

## 3 Results

### 3.1 Clinical features of the MG and non-MG groups

Overall, 104 patients in the MG group accounted for 20.4% of the total patient population, whereas 405 in the non-MG group accounted for 79.6%. No statistically significant differences were observed between the two groups as regards the Masaoka–Koga stage, TNM stage, or surgical resection status (*p* > 0.05). It is noteworthy that R0 resection was the predominant resection status in all cases, with non-R0 resection accounting for 4.8% in the MG group and 3.7% in the non-MG group. The primary reason for R1 resection was the identification of tumor residue on the postoperative incision margin under microscopic examination. R2 resections were performed due to tumor invasion of major vascular structures (superior vena cava, innominate vein, aorta, pulmonary artery) and metastasis to pleura and pericardium, rendering radical resection unfeasible. However, compared to the non-MG group, the MG group exhibited a higher proportion of female patients (*p* = 0.036) and a lower mean age at diagnosis (47.6 ± 12.2 years vs. 51.4 ± 11.9 years, *p* = 0.004). Additionally, CT images demonstrated a smaller mean tumor diameter in the MG group (5.5 ± 2.3 cm vs. 6.2 ± 2.8 cm; *p* = 0.012). Surgical approaches differed significantly between the two groups, with open surgery using median sternal incision being predominant in the MG group, whereas thoracoscopic minimally invasive surgery was more common in the non-MG group (*p* = 0.001). Regarding WHO histopathological types, in the MG group, B2 type, accounting for 42.3%, was the most common, while in the non-MG group, AB type accounting for 41.0% was the most common (*p* < 0.001). Postoperative adjuvant therapy (including chemotherapy, radiotherapy, and targeted therapy) was administered to 50 patients (48.1%) in the MG group and 151 (37.3%) in the non-MG group. Patients with thymomas combined with MG received postoperative adjuvant therapy more than those without MG (*p* = 0.045).

After the PSM, 96 pairs of patients were successfully matched. Baseline clinical data showed no statistically significant differences between the two groups (*p* > 0.05). Comparability was achieved regarding variables across the groups by eliminating the influence of confounding factors. Baseline characteristics of patients are presented in Table 1.

**Table 1:** Clinical characteristics of patients with myasthenia gravis combined with thymoma and thymoma alone before and after matching by PSM method.

Clinicopathological features	Overall					After evaluation using PSM method				
	MG (*n* = 104)		Non-MG (*n* = 405)		*p* value	MG (*n* = 96)		Non-MG (*n* = 96)		*p* value
Sex, *n* (%)					0.036					0.238
Male	44 (42.3%)		218 (53.8%)			42 (43.8%)		34 (35.4%)		
Female	60 (57.7%)		187 (46.2%)			54 (56.2%)		62 (64.6%)		
Age (years)	47.6 ± 12.2		51.4 ± 11.9		0.004	48.3 ± 11.7		49.6 ± 11.3		0.420
Tumor size (cm)	5.5 ± 2.3		6.2 ± 2.8		0.012	5.6 ± 2.3		6.1 ± 2.2		0.155
Approach, *n* (%)					0.001					0.111
MIS	50 (48.1%)		264 (65.2%)			49 (51.0%)		38 (39.6%)		
Sternotomy	54 (51.9%)		141 (34.8%)			47 (49.0%)		58 (60.4%)		
Masaoka-Koga stage, *n* (%)					0.196					0.274
I	47 (45.2%)		197 (48.6%)			43 (44.8%)		46 (47.9%)		
II	35 (33.6%)		158 (39.0%)			33 (34.4%)		39 (40.7%)		
III	22 (21.2%)		50 (12.4%)			20 (20.8%)		10 (10.4%)		
WHO histological classification, *n* (%)					<0.001					0.836
A	2 (1.9%)		38 (9.4%)			2 (2.1%)		2 (2.1%)		
AB	29 (27.9%)		166 (41.0%)			28 (29.2%)		35 (36.5%)		
B1	24 (23.1%)		78 (19.3%)			22 (22.9%)		21 (21.9%)		
B2	44 (42.3%)		95 (23.5%)			39 (40.6%)		35 (36.5%)		
B3	5 (4.8%)		28 (6.9%)			5 (5.2%)		3 (3.1%)		
TNM stage, *n* (%)					0.337					0.092
I	86 (82.7%)		357 (88.1%)			80 (83.3%)		88 (91.7%)		
II	13 (12.5%)		32 (7.9%)			11 (11.5%)		3 (3.1%)		
III	5 (4.8%)		16 (4.0%)			5 (5.2%)		5 (5.2%)		
Completeness of resection, *n* (%)					0.815					1.000
R0	99 (95.2%)		390 (96.3%)			91 (94.8%)		92 (95.8%)		
R1/R2	5 (4.8%)		15 (3.7%)			5 (5.2%)		4 (4.2%)		
Adjuvant therapy, *n* (%)	50 (48.1%)		151 (37.3%)		0.045	45 (46.9%)		47 (49.0%)		0.773

MG, myasthenia gravis; MIS, minimally invasive surgery; PSM, propensity score matching; WHO, World Health Organization.

### 3.2 MG in thymomas: a protective or risk factor?

By the follow-up deadline, 8 patients had died from thymoma, including 4 (3.84%) in the MG group and 4 (0.99%) in the non-MG group. Overall, 28 patients experienced recurrence or metastasis, with 10 (9.62%) in the MG group experiencing recurrence; 7, 2, and 1 experienced local, regional, and distant recurrences, respectively. In the non-MG group, 18 patients (4.44%) were found with recurrence: 7, 7, and 4 experienced local, regional, and distant recurrences, respectively. In the MG group, a postoperative MG response was observed in 79 patients (76%), whereas no response was observed in 25 (24%).

**Figure 2:**
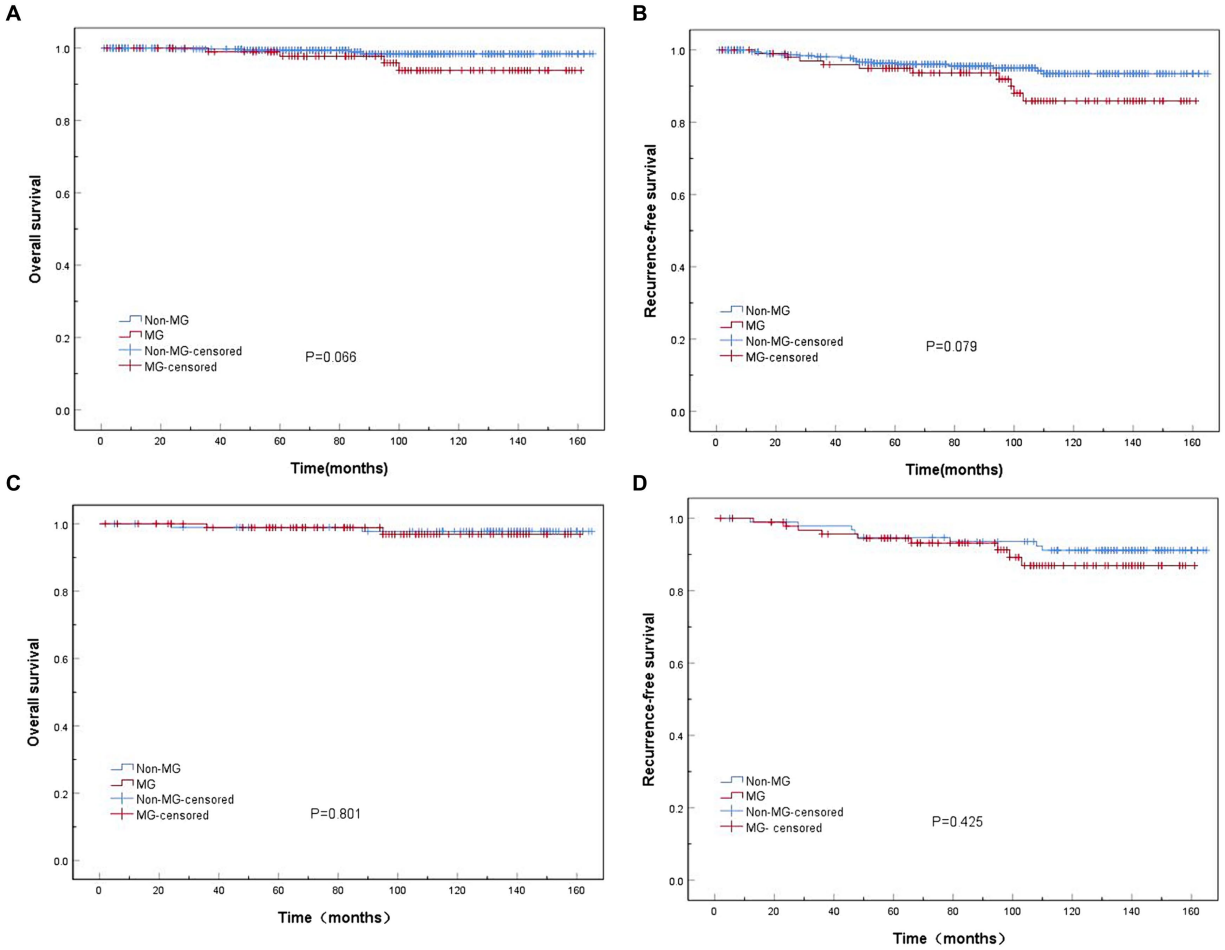
Kaplan–Meier overall survival (OS) and recurrence-free survival (RFS) curves in patients in the myasthenia gravis (MG) and non-MG groups. **(A)** No significant difference in overall survival is observed between the MG and non-MG groups (*p* = 0.066). **(B)** No significant difference in RFS is observed between the MG and non-MG groups (*p* = 0.079). **(C)** No significant difference in overall survival is observed between the MG and non-MG groups (*p* = 0.801). **(D)** No significant difference in RFS is observed between the MG and non-MG groups (*p* = 0.425). **(A,B)** Before matching; **(C,D)** after matching.

Before PSM, the 10-year cumulative survival rates in patients with thymoma were approximately 93.8 and 98.4% in the MG and non-MG groups, respectively. Similarly, the 10-year cumulative rates of RFS were 85.9% in patients with MG and 93.4% in those without MG; however, no significant differences were observed between the two groups (*p* > 0.05) (Figures 2A,B). After PSM, the 10-year cumulative survival rates in patients with thymoma in the MG and non-MG groups were 96.9 and 97.7%, respectively, whereas the corresponding RFS rates were 86.9 and 91.1%, respectively. No statistically significant differences were observed between the two groups in terms of survival curves (*p* > 0.05), respectively (Figures 2C,D).

### 3.3 Prognostic factors for thymoma

The univariate regression analysis for RFS of 509 patients with thymoma after surgery revealed that the WHO histopathological classification (hazard ratio [HR] = 0.431, 95% confidence interval [CI]: 0.199–0.935, *p* = 0.033), Masaoka–Koga stage (HR = 3.561, 95%CI: 1.668–7.605, *p* = 0.001), TNM stage (HR = 2.882, 95%CI: 1.269–6.546, *p* = 0.011), resection status (HR = 4.104, 95%CI: 1.422–11.845, *p* = 0.009), and postoperative adjuvant therapy (HR = 4.672, 95%CI: 1.422–11.845, p = 0.001) may be associated with tumor recurrence following thymoma surgery. Factors demonstrating statistically significant differences in the aforementioned univariate analysis were included in the multivariate Cox-proportional hazard models for further analysis, which showed that Masaoka–Koga stage (HR = 3.307, 95%CI: 1.012–10.812, *p* = 0.048) and postoperative adjuvant treatment (HR = 3.712, 95%CI: 1.491–9.239, *p* = 0.005) were independent predictors of postoperative thymoma recurrence risk. Univariate and multivariate regression analyses for RFS in 509 patients with thymoma after surgery are presented in Table 2.

**Table 2:** Univariate and multivariate regression analyses of recurrence-free survival in 509 patients with thymoma after surgery.

Variable	Univariate analysis			Multivariate analysis			
	HR	95%CI	*p*	HR	95%CI		*p*
Sex (male vs. female)	1.099	0.524–2.306	0.802				
Age, years (≤50 vs. >50)	0.837	0.399–1.758	0.638				
Tumor size (≤5 vs. >5)	1.082	0.514–2.280	0.836				
Approach (MIS vs. sterontomy)	0.970	0.448–2.103	0.939				
Masaoka stage (I + II vs. III)	3.561	1.668–7.605	0.001	3.307	1.012–10.812		0.048
WHO histological Classification (A + AB vs. B1 + B2 + B3)	0.431	0.199–0.935	0.033	0.626	0.283–1.383		0.247
TNM stage (I vs. II + III)	2.882	1.269–6.546	0.011	0.564	0.137–2.313		0.426
Completeness of resection (R0 vs. R1/R2)	4.104	1.422–11.845	0.009	2.234	0.595–8.393		0.234
Adjuvant therapy (Yes vs. No)	4.672	1.894–11.526	0.001	3.712	1.491–9.239		0.005
Myasthenia gravis (Yes vs. No)	0.146	0.020–1.075	0.059				

CI, confidence interval; HR, hazard ratio; MIS, minimally invasive surgery; WHO, World Health Organization.

**Figure 3:**
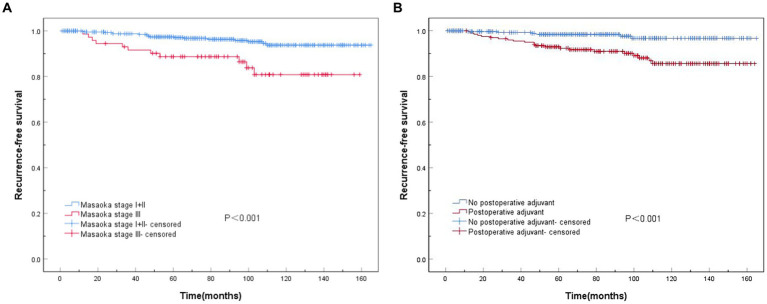
Recurrence-free survival (RFS) curves associated with Masaoka staging and postoperative adjuvant therapy. **(A)** Statistically significant differences in RFS among Masaoka stages I, II, and III (*p* < 0.001). **(B)** Significant differences are observed in RFS with or without postoperative adjuvant therapy (*p* < 0.001).

Survival analysis demonstrated a significantly lower risk of postoperative recurrence in Masaoka–Koga stages I and II than in stage III (*p* < 0.001) (Figure 3A). Additionally, patients who received postoperative adjuvant therapy exhibited a significantly higher risk of recurrence than those who did not receive adjuvant therapy (p < 0.001) (Figure 3B).

## 4 Discussion

MG pathogenesis involves acquired antibody-mediated dysfunction of the neuromuscular junction. Thymic abnormalities such as thymoma and thymic hyperplasia are associated with the occurrence of MG (13). Among patients with thymoma, MG is the most common paraneoplastic neurological disease, affecting approximately 30–50% of these patients (2–4). In this retrospective study, we analyzed the clinical and pathological data of 509 patients with thymomas. The incidence of MG was 20.4%, which was lower than that previously reported in the literature, owing to our hospital’s focus on the early-stage thymoma treatment. Thymectomy has been shown to effectively improve the clinical symptoms and prognosis in patients with MG (14, 15). For patients with thymomas combined with MG, thymectomy is considered the preferred approach for achieving stable disease control and has demonstrated superior efficacy compared to drug therapy alone (16). In this study, the MG remission rate in patients with thymoma after resection was 76.0%. Furthermore, postoperative adjuvant therapy did not significantly affect the efficacy of MG treatment. Previous studies have reported an incidence rate of 1.0–28% for developing MG after surgery among patients who were not initially diagnosed with MG before thymoma resection (17, 18). These findings suggest that while thymoma is a known cause of MG, other underlying mechanisms are involved in its occurrence and progression. The immunoregulatory function of the thymus relies heavily on CD4 + T lymphocytes (19), and it has been proposed that this immune regulation is compromised following thymoma resection, leading to heightened B-lymphocyte activity and increased antibody production (20). Additionally, some studies have suggested that the presence of an ectopic thymus or microthymoma within the adipose tissue surrounding the main tumor may contribute to postoperative myasthenia or myasthenic crisis (18). Therefore, extended thymectomy should be considered as standard care for patients with both thymoma and MG, not only from an oncological perspective but also as a strategy to optimize therapeutic outcomes for MG.

Patients with thymoma combined with MG typically are10 years younger than those with thymoma alone. This study observed a statistically significant age difference between the two groups. The most prevalent occurrence of thymoma combined with MG was found in patients aged 41–50, while that of thymoma alone was in patients aged 51–60. Gender distribution analysis revealed that the majority of patients with thymoma combined with MG were female (57.7%), whereas the majority of those with thymoma alone were male (53.8%). Furthermore, our findings demonstrated that the maximum tumor diameter in cases of thymoma combined with MG was significantly smaller than that observed in patients solely diagnosed with thymoma, which is consistent with the relevant literature reports (21). Possible explanations include the following: symptoms associated solely with thymomas are often inconspicuous and are generally detected through physical examinations when tumors enlarge and compress the surrounding tissues. Previous studies have suggested a potential association between the presence of MG and histological subtypes of thymomas; specifically, higher incidences of MG have been reported among patients exhibiting aggressive tissue types, such as B2 and B3, compared to those exhibiting the A, AB, or B1 subtype (22, 23). Our study revealed that AB type was the most common pathology among patients exclusively diagnosed with thymomas, constituting approximately 50.4% of cases. Conversely, B2 type emerged as the predominant pathology in cases involving both MG and thymomas. Notably, the incidence of MG was lower in subgroups A and AB than in subgroup B2.

However, the effect of MG on the prognosis in patients with postoperative thymoma remains controversial. MG was initially considered an unfavorable prognostic factor for thymomas because of perioperative myasthenic crisis and increased perioperative mortality (24). However, several studies have demonstrated a favorable prognosis in patients with thymoma combined with MG. For instance, one study reported 5- and 10-year survival rates of 90 and 86%, respectively, in patients with thymoma combined with MG, compared to 85 and 75%, respectively, in those with thymoma alone (*p* = 0.046) (25). Another study also revealed a higher 10-year survival rate among patients with MG-associated thymoma (82%) than among those without MG (62%) (*p* = 0.001) (26). The contradictory relationship between the prognosis in patients with thymoma and MG may be attributed to the limitations of clinical studies, such as small sample sizes, a low number of endpoint events, long follow-up periods, and challenges in case enrollment for prospective randomized trials. In non-randomized clinical studies, the unbalanced distribution of confounding factors between the observation and control groups often affects the correct estimation of treatment effects. To address these comprehensively, we employed PSM to integrate multiple confounding variables into a single variable (propensity score). By effectively balancing the propensity scores of the two comparison groups, PSM enables to mitigate the influence of confounding factors and enhances both scientific rigor and accuracy when drawing conclusions from non-randomized clinical studies. In this study, after PSM matching, the baseline clinical data of the patients in both groups were similar. The 10-year survival rates in patients in the MG (*n* = 96) and non-MG (*n* = 96) groups were 96.9 and 97.7%, respectively, and the corresponding 10-year RFS rates were 86.9 and 91.1%, respectively. Notably, no statistically significant difference was observed in the survival curves between the two groups (*p* > 0.05). These results indicate that prognosis in patients with thymoma was not affected by its combination with MG. We employed two statistical methods, the Cox proportional risk model and the PSM method, to evaluate the impact of MG on patients with thymomas’ prognosis, both of which consistently demonstrated that MG did not affect the prognosis of patients with thymoma, thereby strengthening our findings.

Thymomas are relatively indolent tumors with low malignant potential. The most common route of metastasis is regional pleural space implantation, whereas distant metastasis outside the chest is rare, resulting in a favorable long-term prognosis. Historically, the Masaoka stage has been considered a crucial prognostic factor for predicting treatment outcomes and assessing postoperative prognosis in patients with thymoma. Multivariate analysis of clinical data from 509 patients who underwent thymoma surgery revealed that Masaoka–Koga stage (HR = 3.307, 95%CI: 1.012–10.812, *p* = 0.048) independently influenced RFS as an important prognostic factor following thymoma surgery. Postoperative adjuvant therapy (HR = 3.712, 95%CI: 1.491–9.239, *p* = 0.005) was identified as an unfavorable prognostic factor, consistent with previous findings (12, 27, 28). Previous studies demonstrated that achieving R0 resection was a crucial determinant of prognosis in patients with thymoma (29, 30). In this study, univariate analysis revealed significantly improved RFS in patients who underwent R0 resection compared with those who did not. However, multivariate analysis indicated that R0 resection did not independently affect RFS, possibly because of the limited number of non-R0 resection samples and shorter follow-up duration. Therefore, early diagnosis of patients with thymoma is imperative to develop the most appropriate treatment plan. For early-stage patients, optimal outcomes can be achieved using minimally invasive approaches and complete resection.

Currently, the widely used Masaoka–Koga staging system is based solely on the 5-year follow-up results of <100 patients from a single center (31). Although this staging system has been clinically validated, it inadequately reflects specific clinical conditions, because it primarily focuses on local invasion of the primary tumor while neglecting local lymph node metastasis. To address these limitations, the International Association for the Study of Lung Cancer and the International Thymic Malignancy Interest have proposed significant changes in the ninth TNM stage classification of thymus tumors compared to the Masaoka–Koga staging system. For instance, tumor size has been included in the T stage for the first time with a cut-off value of 5 cm limited to thymus tumors and surrounding fat; tumors with a diameter ≤ 5 cm are classified as T1a stage, whereas those >5 cm are categorized as T1b stage. Additionally, Stage II in the Masaoka-Koga classification was redefined as Stage I, while minimal modifications were made to the Stage III and IV classifications. The new TNM staging system may better reflect the correlation between thymoma staging, clinical treatment outcomes, and prognosis, thereby facilitating improved clinical research (11). Univariate regression analysis demonstrated that RFS was significantly better in patients with TNM stage I than in those classified as stage II or III. However, multivariate regression analysis revealed that the TNM stage was not an independent prognostic factor in these patients. This could be attributed to the higher proportion (87.2%) of early-stage patients diagnosed within this study cohort and the shorter follow-up duration.

In comparison to previous studies, this study’s strengths lie in its larger sample size and an extended follow-up period, ensuring the robustness of the results. Additionally, the utilization of PSM method effectively balances the distribution of confounding factors among groups and reduces treatment effects bias, thereby enhancing the reliability and consistency of the findings. However, this study had some limitations. First, the clinicopathological data collected spanned a long period, which may have introduced variations because of advancements in medical technology and differences in treatment efficacy for patients with MG. Additionally, although using the PSM method helps control for confounding variables and enhances the reliability of our conclusions, acknowledging that this single-center retrospective study had a relatively small sample size is essential. Therefore, we anticipate a large-scale multicenter clinical retrospective study to validate and reinforce the findings of this study.

In summary, MG is relatively common in patients with thymoma. However, the presence of MG does not independently affect patient prognosis following thymoma surgery, as demonstrated by both the overall analysis and PSM. The Masaoka–Koga stage remains an independent prognostic factor in RFS in patients with thymoma, whereas postoperative adjuvant therapy is a negative prognostic indicator.

## Data availability statement

The original contributions presented in the study are included in the article/supplementary material, further inquiries can be directed to the corresponding author.

## Author contributions

KZ: Conceptualization, Data curation, Methodology, Writing – original draft, Writing – review & editing. YL: Conceptualization, Data curation, Methodology, Writing – original draft, Writing – review & editing. MJ: Conceptualization, Data curation, Methodology, Writing – review & editing. WC: Conceptualization, Data curation, Methodology, Writing – review & editing. JJ: Conceptualization, Data curation, Methodology, Writing – review & editing. ZZ: Conceptualization, Data curation, Methodology, Writing – review & editing. LS: Conceptualization, Data curation, Methodology, Writing – review & editing. JW: Conceptualization, Data curation, Methodology, Writing – review & editing. ZX: Conceptualization, Supervision, Writing – review & editing.
